# Strategic and Holistic Approach to Sustainable Development in Hospitals—Perspectives of Nurse Executives: A Qualitative Study

**DOI:** 10.1155/jonm/5296442

**Published:** 2025-08-19

**Authors:** Seda Tugba Baykara Mat, Hatice Mutlu

**Affiliations:** Faculty of Health Sciences, Istanbul Beykent University, Cumhuriyet District, Beykent, Avalon, Beylikdüzü, Istanbul, Turkey

**Keywords:** hospitals, nurse administrators, organizational policy, qualitative research, sustainable development

## Abstract

**Background:** Sustainability practices in hospitals with international quality certifications were examined within the scope of corporate strategies, regulations, and healthcare accreditation practices based on the experiences of nurse executives.

**Methods:** This descriptive qualitative study focuses on exploring the experience of nurse executives in sustainable practices. Using a snowball sampling method, semistructured in-depth interviews were conducted with nine nurse executives via an online platform between March 2023 and May 2024. The qualitative data obtained were analyzed using the content analysis method.

**Results:** A total of 9 executive nurses were included in the study. Thematic analysis revealed four main themes: Employee Engagement and Awareness, Structured Practices, Strategic Approach, and Greenwashing. Participants emphasized the importance of increasing employee awareness and managing resistance to change. Structured practices included waste and resource management efforts observed in hospitals. The strategic approach theme highlighted the lack of standardized government policies and the need for integration into hospital strategic planning. The greenwashing theme captured concerns over superficial or reputational sustainability efforts. A total of 9 executive nurses were included in the study. Participants had an average age of 41, with 8 years of professional experience and 6 years in managerial roles.

**Conclusion:** The continuity of awareness is crucial for keeping ecoanxiety at acceptable levels and encouraging nurses to contribute to the development of environmentally friendly healthcare policies.

## 1. Introduction

In today's business world, hospitals must incorporate sustainability principles into their healthcare services. These principles involve meeting current needs without jeopardizing the ability of future generations to meet their own needs. Sustainability also includes conserving natural resources, maintaining environmental balance, meeting societal needs, and improving economic well-being.

Sustainable resource management mitigates environmental impact and results in cost savings. Compelling collection, segregation, and disposal of hospital waste at its source reduces health risks and minimizes hospital expenditures [1].

Given the ongoing operational demands of hospitals providing inpatient and outpatient services, significant energy, water, and resources are consumed. Hospitals with high energy demands can achieve substantial environmental improvements through energy efficiency initiatives and a shift toward renewable energy sources [2, 3].

The “Green Hospital” paradigm within the healthcare sector embodies a model characterized by environmentally friendly practices that enhance public and environmental health while ensuring effective resource management. Green hospitals prioritize energy efficiency and incorporate integrated waste reduction and processing facilities. Moreover, these institutions establish green infrastructures to foster a healthier environment for patients, staff, and the broader community [4]. These sustainability practices in the healthcare sector underscore the imperative of incorporating sustainability into corporate strategies and aligning with legislative frameworks that inform these strategies. This integration promotes environmental stewardship and supports the broader goals of public health and operational efficiency within healthcare facilities. Recent literature has proposed various frameworks to support environmentally sustainable practices in healthcare. These models emphasize the integration of green infrastructure, digital transformation, and patient-centered approaches to reduce environmental impact while improving operational efficiency and care quality [5]. Some studies have also analyzed the carbon footprint of surgical procedures, offering critical insights into how environmental impacts can be mitigated [6]. In this context, Wang et al. highlighted several sustainable practices in orthopedic surgery—including waste reduction, energy efficiency, and the use of reusable surgical instruments—which contribute not only to environmental protection but also to operational gains and improvements in the quality of patient care. These approaches demonstrate that sustainability is not merely an environmental issue but also a strategic component of healthcare system performance [7].

These sustainability practices in the healthcare sector underscore the imperative of incorporating sustainability into corporate strategies and aligning with legislative frameworks that inform these strategies [8–10]. This integration promotes environmental stewardship and supports the broader goals of public health and operational efficiency within healthcare facilities.

Government guidelines and incentives are recognized as crucial factors influencing the formulation of sustainability initiatives in contemporary healthcare organizations, both presently and in the foreseeable future [11]. Nurse executives are pivotal in orchestrating and implementing sustainability practices within hospital settings. However, existing literature is sparse regarding their proficiency in sustainability knowledge, their practical implementation experiences, and the challenges they encounter.

This study aims to gain a comprehensive understanding of how sustainability is perceived, implemented, and managed in hospital settings, specifically from the perspective of executive nurses. It focuses on exploring their awareness, the strategies they employ, and the challenges they encounter in integrating sustainability into healthcare operations. With its distinct focus, targeted sample group, and qualitative methodology, the research provides valuable insights that can support sustainable practices and inform healthcare policy, accreditation standards, and strategic planning efforts. In line with this objective, the study poses the following research question: “What are the perspectives of executive nurses working in hospitals accredited with internationally recognized health-specific quality certificates on sustainable hospital practices?”

## 2. Materials and Methods

### 2.1. Study Design

This study employed a descriptive qualitative design, focusing on exploring the experiences of nurse executives in sustainable practices [12]. The reporting of this study adhered to the Consolidated Criteria for Reporting Qualitative Research (COREQ) checklist [13]. Please see Supporting Information 1 for the completed COREQ checklist.

### 2.2. Ethical Approval

Ethical approval for this study was obtained from the Scientific Research and Publication Ethics Committee for Social and Human Sciences of Istanbul Beykent University (approval no: 139134, date: 15/02/2024). The study was conducted in accordance with the ethical standards of the institutional and/or national research committee and with the 1964 Helsinki Declaration and its later amendments. Participation was voluntary, and informed consent was obtained from all individuals included in the study.

### 2.3. Population and Sample Selection in Qualitative Research

In qualitative research, the population selection differs significantly from quantitative approaches, as the aim is not to achieve statistical representativeness of the entire universe. Instead, the emphasis lies on obtaining rich and diverse data that can illuminate the chosen topic deeply. Wallace and Wolf advocate for a purposive sampling strategy in qualitative studies, where the goal is to gather information-rich cases that provide nuanced insights into the phenomenon under investigation [14]. This approach acknowledges that qualitative research does not seek to generalize findings across a population but to uncover the breadth and depth of experiences and perspectives relevant to the research question. Thus, selecting participants is intentionally biased toward individuals or cases that can offer diverse and comprehensive perspectives, ensuring the richness and depth of the qualitative data collected.

### 2.4. Study Population and Inclusion Criteria

The study's population comprised executive nurses employed in hospitals in Istanbul, holding an internationally recognized, health-specific quality accreditation certificate. Executive nurses in this study refer to senior-level registered nurses responsible for unit-level or institutional management, including administrative decision-making, strategic planning, implementation of quality standards, and oversight of clinical operations. The study focused on hospitals located in Istanbul, both Asia and Europe.

### 2.5. Inclusion Criteria

#### 2.5.1. Hospital Criteria

Hospital criteria were defined as follows:

• Located within Istanbul.
• Possesses an internationally recognized, health-specific quality accreditation certificate for a minimum of 4 years.

#### 2.5.2. Participant Criteria

Participant criteria were defined as follows:

• Holds at least a bachelor's degree in nursing.
• Employed at the same institution for a minimum of 3 years.
• Serving as a nurse executive within the same institution for at least two years.
• Willingly volunteers to participate in the study.

### 2.6. Exclusion Criteria

#### 2.6.1. Hospital Criteria

Hospital criteria were defined as follows:

Located outside of Istanbul.

Lacks an internationally recognized, health-specific quality accreditation certificate.

Does not hold an internationally recognized, health-specific quality accreditation certificate for at least 4 years.

#### 2.6.2. Participant Criteria

Participant criteria were defined as follows:

Possesses a bachelor's degree in a field other than nursing.

Employed in the organization for less than 3 years.

Has not served as a nurse executive within the same institution for at least two years.

Declines to volunteer to participate in the study.

This approach ensures that the participants selected for the study possess the relevant qualifications and experience necessary to provide comprehensive insights into sustainable hospital practices within Istanbul's specified context.

The study employed the snowball sampling method, a purposeful sampling technique, to identify executive nurses willing to participate. In this method, participants are deliberately selected based on predefined criteria. As interviews progress and new insights emerge, information accumulates like a snowball effect, aiming to enrich the dataset with diverse cases [11]. The data collection phase in snowball sampling continues until data saturation is achieved through successive interviews conducted in a chain-like manner [15].

In reporting the research findings, direct quotations are utilized to illustrate the investigated phenomenon effectively. This approach aims to present the nuanced perspectives and experiences of the participants accurately and vividly.

During the study, 12 executive nurses employed in hospitals holding international accreditation certificates were initially identified. After careful screening, three participants who did not meet the predefined inclusion criteria were excluded from the study: one nurse who had been an executive nurse for only 1 year and two nurses whose hospital's quality certificate did not extend back at least 4 years.

**Figure 1:**
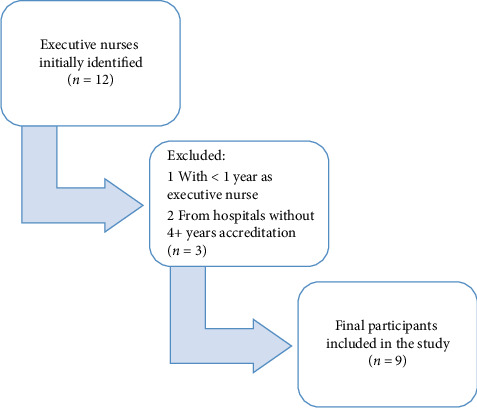
Flowchart showing inclusion and exclusion criteria.

To enhance methodological transparency, a flowchart has been included to illustrate the inclusion and exclusion criteria used during the participant selection process. The flowchart outlines the initial pool of participants, reasons for exclusion, and the final sample included in the study (Figure 1).

Subsequently, planned online individual in-depth interviews were conducted with the remaining nine nurse executives. Each interview session was structured around semistructured interview guides and averaged approximately 50 min. The interviews were designed to explore diverse perspectives and experiences related to sustainable practices in hospital management. Interviews were concluded upon reaching data saturation, indicated by recurring themes and repetitive insights from participants.

All interviews were conducted in Turkish, which allowed participants to share their views in their native language with ease. The data were analyzed in Turkish to preserve the original meaning and nuance. Key excerpts were translated into English for reporting purposes, and bilingual researchers reviewed translations to ensure accuracy.

The selection of nurse executives spanned various clinical areas within their hospitals, ensuring a comprehensive representation of perspectives. Participants were contacted based on recommendations from nurse executives across different hospitals, who facilitated introductions and endorsed their participation in the study.

Overall, this methodological approach aimed to gather rich and detailed insights into the role of sustainability practices in hospital management, as perceived and experienced by seasoned executive nurses.

### 2.7. Data Collection

The qualitative phase of the study involved initial interviews with participants, during which researchers introduced themselves and the study, addressed participant concerns, and aimed to establish trust. Appointments were scheduled at convenient times for participants, and online interviews were conducted via Zoom (Zoom Video Communications, Inc., San Jose, CA, USA) in quiet, private settings chosen by the participants.

Before the interviews, detailed information about the study's purpose, publication process, and informed consent form was provided. Participants were asked to complete the informed consent process before the interviews.

The research team consisted of two female academic researchers, both of whom held PhDs in health sciences. Author 1 is a registered nurse with professional experience in hospital-based clinical care and has received formal training in both basic and advanced qualitative research methodologies. Author 2 previously held managerial roles in hospital quality management systems and brings expertise in healthcare leadership and accreditation processes. Both researchers currently serve as assistant professors at a Faculty of Health Sciences within a foundation university.

Interview sessions commenced with researchers introducing themselves and participants introducing their respective institutions. This initial phase established rapport and created a comfortable atmosphere before transitioning into the structured interview questions.

Researchers meticulously observed participants' responses throughout the interviews, noting any signs of anxiety, emotionality, or frustration conveyed through facial expressions and verbal cues. Moments of silence and pauses were deliberately recorded and subsequently subjected to detailed analysis. These observations aimed to capture nuanced insights and understandings that could enrich the qualitative data analysis.

Individual interviews ranged from a minimum of 52 min to a maximum of 88 min, with an average duration of 66 min among the 9 participants. In total, 594 min of recordings were accumulated from these interviews.

All in-depth interviews were conducted with participants' explicit consent, and recordings were securely stored on the researchers' computers for subsequent analysis. Raw data from the recordings were transcribed verbatim to facilitate thorough analysis. Transcripts were then shared with participants to ensure accuracy and allow them to review and verify the information gathered.

This meticulous process adheres to best practices in qualitative research, aiming to maintain data integrity and transparency while honoring participants' contributions to the study.

### 2.8. Place and Time of the Research

The semistructured individual interviews, planned within the scope of this research, were conducted online using a digital platform between March 2023 and May 2024 in Istanbul.

### 2.9. Data Collection Tools

#### 2.9.1. Personal Information Form

A Personal Information Form was utilized, comprising five questions about the participants' sociodemographic characteristics.

#### 2.9.2. Semistructured Interview Form

The “Semistructured Executive Nurse Interview Form,” developed based on a review of relevant literature, was employed to gather participants' perspectives [16]. This interview form, designed to align with the research objectives, includes seven open-ended questions to explore the participants' demographic characteristics, feelings and behaviors, knowledge, beliefs, and experiences [17–19].

Before commencing the individual in-depth interviews, pilot interviews were conducted with three executive nurses who were not part of the primary study sample. This pilot phase ensured the clarity and effectiveness of the interview questions and format.

**Figure 2:**
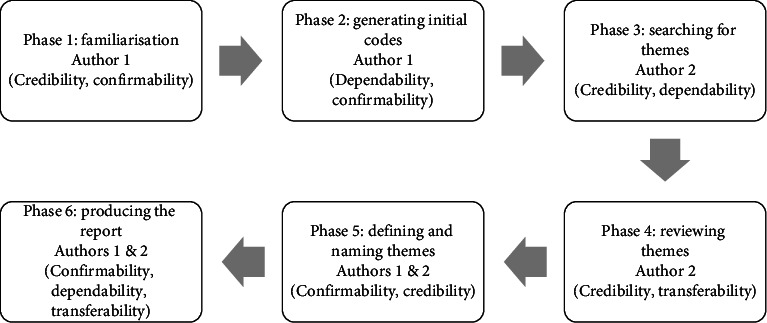
Thematic analysis process, researcher roles, and trustworthiness criteria.

To ensure methodological rigor, the study adopted the six-phase thematic analysis framework proposed by Braun and Clarke [20]. Analytical responsibilities were deliberately distributed between Author 1 and Author 2 to enhance the depth of interpretation and reduce the risk of researcher bias. As illustrated in Figure 2, each analytical phase was aligned with established trustworthiness criteria—credibility, transferability, dependability, and confirmability—based on the framework of Lincoln and Guba [21], thereby strengthening the transparency and validity of the analytical process.

### 2.10. Data Analysis

The content analysis method was employed to analyze qualitative data in this study. Content analysis involves systematically coding the collected data into meaningful categories and organizing these codes into subthemes and overarching themes. These themes encapsulate the scope and essence of the research findings.

### 2.11. Data Analysis Methodology

In this study, content analysis was employed to analyze qualitative data, using the inductive method to generate themes from the coded interview transcripts. The NVivo (QSR International Pty Ltd., Melbourne, Australia) program facilitated this process by systematically coding statements relevant to the research objectives into main and subthemes. The NVivo program was instrumental in organizing and categorizing the information gathered from qualitative research (22). NVivo played a crucial role in organizing, categorizing, and compiling the data, facilitating a comprehensive analysis. Initial readings of all transcripts captured participants' statements aligned with the research objectives, which were subsequently coded into main and subthemes using NVivo.

In the qualitative phase, researcher (Author 1) reviewed transcripts rigorously to establish codes from participants' statements, grouping repeated concepts under corresponding codes for coherence. Data were categorized into subheadings, codes, subthemes, and central themes to ensure thorough organization and analysis. Using an inductive method, themes were systematically developed from interview content, providing a structured analysis that captured participants' perspectives and experiences on the research objectives. The themes and subthemes formed after the analysis were presented to the participants for approval.

## 3. Results

When examining the sociodemographic characteristics of the participants according to the research findings, the average age was found to be 41. Forty-four percent (4 individuals) of the participants were female, with an average professional experience of 8 years and an average of 6 years in managerial positions. Thirty-three percent (33%) of the participants were nursing services managers (NSMs) and 22% were charge nurses (CH). Two participants joined the interviews as assistant nursing services managers (ANSMs). Regarding educational attainment, 56% (five individuals) held a bachelor's degree, 33% (three individuals) were master's or doctoral students, and 11% (one individual) held a doctoral degree (Table 1.)

Table 2 includes the main themes and subthemes identified in the discussions on sustainability. These themes emerged from the study's participants' views and experiences, covering essential topics such as employee engagement and awareness, structured practices, competitive advantage, and greenwashing.

### 3.1. Main Theme 1: Employee Engagement and Awareness

Under this main theme, as discussed in Table 3, participants emphasized the importance of employee engagement in sustainability practices and awareness regarding these issues.

### 3.2. Main Theme 2: Structured Applications

As outlined in Table 4, this main theme encompasses the structured efforts of institutions focused on sustainability. Participants emphasized resource and waste management, evaluating how internal activities contribute to environmental conservation and resource management.

### 3.3. Main Theme 3: Strategic Approach

In Table 5, the participants indicated that sustainability efforts should be integrated within the strategic planning, budgeting, and human resource management framework as a central theme. They also emphasized the importance of regulations and quality management tools for effectively disseminating and standardizing these efforts.

### 3.4. Main Theme 4: Greenwashing

The final theme, “greenwashing,” as discussed in Table 6, highlights participants' criticism that sustainability efforts are conducted not for genuine and ethical purposes but merely for marketing or reputational gains. Participants emphasized that organizations' efforts to appear environmentally friendly are distinct from genuine and effective sustainability practices.

The findings of this study serve as a valuable guide for those developing corporate sustainability strategies, offering an in-depth understanding of the integration and effectiveness of sustainability practices in the business world.

## 4. Discussion

The findings of this study provide significant insights from the perspective of nurse executives into how sustainability practices are perceived and implemented in healthcare institutions. Employee engagement and awareness, structured practices, strategic approaches, and findings grouped under the main themes of greenwashing deepen with various subthemes and are consistent with the literature [22].

Participant views emphasize the necessity of raising employees' awareness about sustainability through training and support. However, as some participants noted, these training and awareness activities are not systematically carried out, and employees need to be sufficiently encouraged to share their opinions. This situation needs to be improved to encourage employee participation. It is crucial to establish a safe environment where employees feel comfortable sharing their ideas and actively participating in sustainability practices [23].

Employee engagement and alignment are critically important for businesses to achieve sustainability goals. The literature offers various perspectives on employees' tendencies to adapt to current practices and resist change. Some studies indicate that employees contribute positively to businesses' sustainability efforts and actively participate in areas such as environmental efficiency. These studies argue that employees' attitudes and behaviors are directly linked to reducing businesses' environmental impacts and strengthening their sustainability efforts [24, 25].

Given the research gap regarding sustainable human resource practices in the healthcare sector, it is critically important to assess the effectiveness and prevalence of this area. There is an emphasis on the need for a detailed examination of the profound impact of sustainable practices on organizational performance. Similarly, strategies must be developed to foster wider adoption of sustainability-focused human resource management practices. In this context, it is highlighted that it is necessary to develop conceptual frameworks that encourage active employee participation in sustainable practices [26]. Given the research gap regarding sustainable human resource practices in the healthcare sector, it is critically important to assess the effectiveness and prevalence of this area. There is an emphasis on the need for a detailed examination of the profound impact of sustainable practices on organizational performance. Similarly, strategies must be developed to foster wider adoption of sustainability-focused human resource management practices. In this context, it is highlighted that conceptual frameworks that encourage active employee participation in sustainable practices are necessary [27]. Nurse executives have expressed their role in facilitating collaboration and integrating processes, considering the impacts of these practices on patient care. The literature encourages hospitals to foster an environmental culture both at the individual and institutional levels to establish a sustainability culture [28]. Otherwise, it is stated that organizations will only be able to achieve sustainability goals with their employees [26].

Another main theme nurse executives emphasize is the cost-effective management of resources examined under structured practices. Hospitals are under significant pressure to achieve cost savings, increase operational efficiency, and maintain service capacities [29]. These challenges necessitate the effective management of hospitals and the consideration of the corporate economic dimension regarding sustainability. Optimizing hospital services, improving processes, expanding into new markets, and ensuring sustainable financial returns are crucial [30]. On the other hand, nurse executives have pointed out that waste management is an indispensable practice. As is well known, hospitals are enterprises that generate high levels of hazardous waste [31]. To ensure environmental sustainability, hospitals must integrate renewable resources and waste management systems into their corporate governance under sustainable policies. To ensure environmental sustainability, hospitals must integrate renewable resources and waste management systems into their corporate governance under sustainable policies. This will reduce environmental impacts and give them a competitive advantage [32]. Under the theme of Strategic Approach, the necessity of addressing sustainability practices from a strategic perspective in collaboration is emphasized.

However, many participants note deficiencies and highlight the need for a specialized human resource structure dedicated to sustainability. Compared with international practices, sustainability in Turkish hospitals appears more voluntary and less systematically integrated. For instance, in Lithuania, a structured sustainability framework based on 57 indicators is implemented through national health strategies [33]. Similarly, centralized pharmaceutical care systems in Italy ensure cost efficiency and institutional consistency [34]. A recent qualitative study conducted in low- and middle-income countries emphasized that hospital sustainability efforts, particularly in antimicrobial stewardship, are frequently linked to accreditation and regulatory compliance mechanisms, which help institutionalize these practices as part of standard healthcare governance [35]. These findings highlight how national policy environments and institutional structures influence the depth and integration of sustainability practices across health systems.

It is stated that hospitals need to structure their sustainability efforts in line with strategic plans and budgets. Faced with ongoing budget deficits, literature underscores that it is increasingly difficult for an organization's leadership to garner sufficient support and participation for such a role unless committed to an environmental sustainability program [36]. Therefore, establishing practices based on standards paves the way for leveraging the existing workforce with optimal efficiency. In this study, participants summarized this as “working collaboratively under a shared vision.” Nurse executives are in the best position to share their knowledge and expertise about the healthcare sector to enhance the system's performance, improve the management process of practices, and provide a competitive advantage [37, 38]. To enhance the effectiveness of environmental sustainability strategies, it is recommended that dedicated full-time experts be responsible for continuously monitoring, evaluating, and developing new strategies. This approach can strengthen these strategies' continuity and implementation success, leading to positive outcomes [20]. For example, case studies on the success of energy-saving strategies in hospitals in the Middle East and North Africa recommend the adoption of ISO 50001 energy management systems to overcome energy management challenges [39]. Based on the findings, sustainability should be addressed holistically within existing accreditation standards specific to healthcare services. The literature indicates that accreditation standards enhance the effectiveness of waste management in hospitals [40]. In this context, while standards support environmental health and nature, thus contributing to sustainable development, no specific criterion within accreditation standards encompasses all targets. This could be addressed as a topic for research and improvement. The literature suggests that the widespread use of artificial intelligence, wearable devices, e-health, telemedicine, and remote care, as well as emerging technologies, genetic studies, and the economic pressures on healthcare delivery, will drive the development of new care models and the structuring of sustainable healthcare systems, including achieving the sustainable development goals (SDGs). Therefore, accreditation needs to be aligned accordingly in this context [41]. It is emphasized that government guidelines and incentives are crucial elements in planning future sustainability initiatives in healthcare facilities [42]. This situation is essential for fostering a common management approach across all organizations. Such systems are seen as essential tools to support SDGs.

In healthcare services, sustainable practices must be redefined to include both environmental and social dimensions and must be measurable. In this sense, each process should be aligned with measurable standards within the context of quality improvement initiatives, with the necessity of periodically monitoring different elements of sustainable value [43]. In addition, the literature notes that integrating sustainable processes into quality improvement efforts is a practical way to facilitate application and development steps and address challenges related to collaboration and alignment [44]. Participatory nurse executives' views regarding establishing standards align with those found in the literature, and their recommendations for a regulatory role are directed toward government agencies. The literature argues that the transparent publication of reports prepared for such audits by dedicated units enhances accountability [45]. Moreover, this transparency can be interpreted as a significant step toward preventing greenwashing practices.

The main theme emerging from this study is greenwashing practices. Nurse executives have used expressions such as “making it seem like it is” and “a marketing technique” regarding current practices. Greenwashing can be defined as conveying a false or misleading impression that a product, service, or process is environmentally friendly or preferable compared with alternatives [46]. Greenwashing practices, widely observed across many sectors, are often carried out solely for marketing purposes rather than genuinely achieving sustainability goals. They are used to gain a reputation rather than making meaningful progress toward sustainability objectives [47]. In other words, environmental awareness has become a part of marketing and trendy strategies within the industry.

One striking finding in the results was that hospital sustainability was predominantly considered within environmentally friendly practices, with little awareness or knowledge among employees regarding SDGs and objectives. Consequently, practices addressing all aspects of sustainable development must become an integral part of business strategies as a global imperative. Literature review reveals that studies predominantly associate sustainability with environmentally focused practices such as green hospitals, emphasizing their role in supporting environmental conservation [48, 49]. However, there needs to be more comprehensive research linking sustainability to all 17 SDGs as an integral part of strategy. This gap presents an opportunity for future research to delve deeper into the topic.

It provides an essential roadmap supported by academic literature for more effective implementation of hospital sustainability practices [22, 50]. Considering aspects such as increasing employee participation and awareness, approaching structured practices with a more strategic perspective, and preventing greenwashing practices can lead to more effective attainment of sustainability goals in the healthcare sector. This study is expected to guide further enhancement and expansion of sustainability practices in the healthcare industry. The themes identified in this study align closely with several United Nations SDGs, underscoring the relevance of hospital-based sustainability practices within global sustainability agendas. The theme of employee engagement and awareness directly corresponds to SDG 8 (Decent Work and Economic Growth), emphasizing empowering health professionals and ensuring inclusive, participatory work environments. Structured Practices, which include institutional waste and resource management efforts, reflect the principles of SDG 12 (Responsible Consumption and Production), aimed at reducing environmental impact through efficient operations. The Strategic Approach theme aligns with SDG 3 (Good Health and Well-being) and SDG 13 (Climate Action), highlighting the need to integrate environmental sustainability into health system governance and long-term planning. Finally, greenwashing is also related to SDG 12, as it exposes the disconnect between reported and actual sustainability performance, pointing to the need for transparency, accountability, and genuine institutional commitment in achieving sustainable production and service models in healthcare.

Despite its contributions, this study has several limitations. First, it is limited to the views of nurse executives working in internationally accredited hospitals in Istanbul and participating voluntarily. Second, the geographical focus of the study on Istanbul may limit the applicability of the results to other regions with different institutional or regulatory environments. Third, the study relies solely on qualitative data obtained through interviews. While this approach was appropriate for exploring participants' perceptions and experiences regarding sustainability practices, it did not allow for the inclusion of observational or documentary evidence such as hospital audit reports, energy consumption records, or waste management logs. The absence of such institutional data limits the ability to compare or validate participants' statements with objective sustainability performance metrics. Future research should integrate qualitative and quantitative data sources, such as hospital sustainability indicators, to provide a more comprehensive and triangulated understanding of sustainability efforts in healthcare settings.

## 5. Conclusion

This study explored sustainability practices in healthcare through the perspectives of nurse executives, yielding critical insights into how these practices are shaped, implemented, and challenged in hospital settings. The results highlighted the need for increased employee engagement, structured and standardized organizational practices, and a strategic approach to resource and waste management. The findings also highlighted the need for greater transparency and accountability in reporting sustainability efforts, highlighting the risk of greenwashing. Based on the findings of the study, we recommend six key actions: (1) strengthen sustainability education programs, (2) involve healthcare personnel early in change processes to reduce resistance, (3) allocate dedicated resources and budgets for sustainability initiatives, (4) include sustainability goals in strategic plans and performance evaluations, (5) ensure continuous monitoring and public disclosure of sustainability results, and (6) make institutional sustainability practices more systematically implemented, aligned with global standards and legislation developed in line with the SDGs and also mandatory. This study contributes to the existing literature by contextualizing sustainability practices in internationally accredited hospitals. It highlights the strategic role of nurse executives in implementing and advocating for sustainability. The findings also serve as a practical roadmap for healthcare policymakers, hospital administrators, and accreditation bodies aiming to integrate sustainable practices within healthcare systems. Future research should consider incorporating quantitative hospital sustainability metrics—such as energy usage, waste volumes, and resource efficiency ratios—alongside qualitative interviews. Including documentary analysis and observational data could enhance the triangulation of findings and offer a more comprehensive evaluation of sustainability practices.

## Figures and Tables

**Table 1:** Characteristics of participants.

Participant	Age	Professional experience (years)	Current position (years)	Education level
Participant 1	38	12	CH/5	Bachelor's degree
Participant 2	39	10	CH/9	Master's level student
Participant 3	42	5	ANSM/4	Bachelor's degree
Participant 4	40	11	NSM/10	Master's level student
Participant 5	40	6	CH/4	Bachelor's degree
Participant 6	48	6	NSM/8	Doctoral degree
Participant 7	46	10	NSM/6	Doctoral degree
Participant 8	36	8	ANSM/5	Bachelor's degree
Participant 9	42	5	NSM/4	Master's/Doctoral level student

**Table 2:** Themes and subthemes related to sustainability experiences of executive nurses.

Themes	Subthemes
Employee engagement and awareness	Awareness
	Employee engagement and adaptation
	Resistance to change

Structured applications	Resource management
	Waste management

Strategic approach	Corporate strategies
	Collaboration and integration
	Standardization

Greenwashing	

**Table 3:** Subthemes and participant views on employee engagement and awareness.

*Subtheme: 1.1. Awareness*	
Description	Nurse executives conveyed that integrating act education, awareness, and practical applications is essential for fostering a sustainability culture within healthcare institutions. This approach contributes significantly to achieving sustainability goals by both staff and the institution.
Participant views	“Due to untrained and unaware personnel, adaptation problems may occur. A culture can be created through in-service training.” (Participant 1/CH-38)
	“They conducted a short survey in the hospital to assess the level of knowledge about proper waste segregation in all departments. Based on the survey results, they provided additional training in the areas with the most knowledge gaps. This was very beneficial.” (Participant 2/CH-39)
	“In my institution, we have a social activity platform. We have recycling projects that increase our awareness.” (Participant 1/CH 38)
	“As part of staff awareness efforts, training can be organized, and brochures and posters can be used to enhance visual memory.” (Participant 3/NSM-42)

*Subtheme: 1.2. Employee participation and cohesion*	
Description	“When we succeed in these types of initiatives, you can feel the team spirit. We do a very demanding job. Ensuring the continuity of our employees is actually our top sustainability goal. Their commitment and organizational socialization should be among our top priorities.” (Participant 6/NSM-48)
	“It is forbidden to deviate from environmental compliance practices. If our ideas are not problematic for them and do not cause issues in their documents, procedures, or instructions, our ideas are implemented, but we are not constantly asked for our input.… My colleagues are very reluctant to express their opinions when something is at stake, whether it involves a change or a new initiative.” (Participant 4/NSM-40)

*Subtheme: 1.3. Resistance to change*	
Description	Nurse executives noted that employees exhibit resistance to sustainability initiatives, with this resistance becoming more pronounced due to the reluctance of long-term employees to change their work habits.
Participant views	“Changing habits is always challenging and requires time.” (Participant 3/NSM-42)
	“People resist accepting new practices because they have become accustomed to the existing order and have developed routines in their work life. This resistance is more pronounced among individuals working for many years.” (Participant 4/NSM-40)

**Table 4:** Subthemes and participant views on structured applications.

*Subtheme: 2.1. Resource management*	
Description	Participants discussed the presence of sustainability-focused activities in the hospitals where they work. They emphasized that these activities contribute to the conservation of natural resources and enhance institutional resource management.
Participant views	“Transitioning to a digital operating system, we aimed to reduce paper waste and allocate the time healthcare staff previously spent on paperwork to patient care, thereby providing patient-centered services.” (Participant 1/CH-38)
	“Material procurement needs to be integrated into institutional decisions and policies to ensure continuity.” (Participant 5/CH-40)
	“They have implemented sensor-equipped faucets to use water more efficiently. An air conditioning system was introduced to maintain standard room temperatures. In addition, a system was established to generate the electricity used in our hospital. These measures are also undertaken to provide a cost advantage.” (Participant 2/CH-39)

*Subtheme: 2.2. Waste management*	
Description	Participants indicated that effective practices are in place for the proper segregation, collection, storage, and disposal of waste at the source. They noted that these practices reduce environmental impacts and hold significant importance for cost management.
Participant views	“There is now serious measurement of waste. We track these items meticulously. We must monitor them for cost purposes.” (Participant 7/NSM-46)
	“Waste bins are labeled. The labels detail which type of waste should be placed in each bin.” (Participant 3/ANSM-42)
	“Training employees on waste management and conducting related inspections are highly effective for ensuring compliance.” (Participant 8/ANSM-36)

**Table 5:** Subthemes and participant views on strategic approach.

*Subtheme: 3.1. Standardization*	
Description	Participants indicated that the government should establish standards for sustainability-focused efforts and environmentally friendly initiatives, such as green hospitals, to be implemented uniformly across private and public healthcare institutions. These standards should be considered criteria within quality management models.
Participant views	“The government's involvement in ensuring that sustainability and green hospital practices are adopted across all hospitals, whether private or public, is crucial. The process is not managed effectively due to the lack of a standard. (Participant 9/NSM -42)
	“I believe that sustainability practices are left to the initiative of the institution's owner and managers in this country.” (Participant 8/ANSM-36)
	“Quality standards require no mandatory and inspected situations.” (Participant 9/NSM-42)

*Subtheme: 3.2. Corporate strategies*	
Description	Participants indicated that sustainability practices should be an integral part of the institution's strategic plan and that resources should be allocated within the framework of strategic human resource management and budgeting approaches.
Participant views	“There is nothing specific about sustainability in the hospital's strategic plans. They indicate that they will continue to improve existing practices, but I am not sure how much will be achieved.” (Participant 6/NSM-48)
	“Unfortunately, we do not have a dedicated team or budget specifically for sustainability.” (Participant 7/NSM-46)

*Subtheme: 3.3. Collaboration and integration*	
Description	Participants' views emphasize the importance of integrating sustainability practices within hospitals. The necessity of adhering to established standards in technical systems such as heating and cooling, as well as the need for collaboration with field personnel, is highlighted. In addition, attention is drawn to the significance of incorporating sustainability measures into patient care practices, considering their direct impact on patients.
Participant views	“You cannot arbitrarily change systems like heating or cooling; for example, the refrigerator temperature is fixed. These are situations where we must collaborate with field personnel.” (Participant 6/NSM-48)
	“Every step toward sustainability must be integrated into patient-care practices, as we work in healthcare and must consider the patient. This requires comprehensive collaboration from top to bottom.” (Participant 9/NSM-42)

**Table 6:** Subthemes and participant views on greenwashing.

*Main theme 4: Greenwashing*	
Description	Participants' views reveal that many organizations in Turkey superficially adopt sustainability practices due to popularity or competition. However, it is emphasized that actions focused solely on outward appearance do not constitute genuine sustainability strategies.
Participant views	“In Turkey, many organizations adopt these practices because they are popular. Many organizations say ‘they have done it, so I should do it too', based on competitive environment.” (Participant 2/CH-39)

## Data Availability

The data that support the findings of this study are available from the corresponding author upon reasonable request. Due to the nature of qualitative data and confidentiality agreements with participants, the full transcripts are not publicly available.

## Supporting Information

The Supporting Information attached to this manuscript includes the Consolidated Criteria for Reporting Qualitative Research (COREQ) checklist. This checklist was completed to ensure the transparency and comprehensiveness of the qualitative components of this study, in alignment with established reporting standards.
