# Supplementary material for: Strategic and Holistic Approach to Sustainable Development in Hospitals—Perspectives of Nurse Executives: A Qualitative Study
Source: J Nurs Manag. 2025 Aug 19;2025:5296442. doi: 10.1155/jonm/5296442 (PMC12380513; doi:10.1155/jonm/5296442)
Supplement: Supporting Information — Additional supporting information can be found online in the Supporting Information section. [file 5296442.f1.docx]

**Consolidated criteria for reporting qualitative studies (COREQ): 32-item checklist**

| **Page No** | **Item** | **Guide questions/description** |  |  |
| --- | --- | --- | --- | --- |
| **Domain 1: Research team and reflexivity** |  |  |  |  |
| Personal Characteristics |  |  |  |  |
| 10 | Interviewer/facilitator | Which author/s conducted the interview or focus group? |  |  |
| 10 | Credentials | What were the researcher's credentials? *E.g. PhD, MD* |  |  |
| 10 | Occupation | What was their occupation at the time of the study? |  |  |
| 10 | Gender | Was the researcher male or female? |  |  |
| 10 | Experience and training | What experience or training did the researcher have? |  |  |
| Relationship with participants |  |  |  |  |
| 11 | Relationship established | Was a relationship established prior to study commencement? |  |  |
| 11 | Participant knowledge of the interviewer | What did the participants know about the researcher? e*.g. personal goals, reasons for doing the research* |  |  |
| 11 | Interviewer characteristics | What characteristics were reported about the interviewer/facilitator? e.g. *Bias, assumptions, reasons and interests in the research topic* |  |  |
| **Domain 2: study design** |  |  |  |  |
| Theoretical framework |  |  |  |  |
| 5 | Methodological orientation and Theory | What methodological orientation was stated to underpin the study? *e.g. grounded theory, discourse analysis, ethnography, phenomenology, content analysis* |  |  |
| Participant selection |  |  |  |  |
| 6-7 | Sampling | How were participants selected? *e.g. purposive, convenience, consecutive, snowball* |  |  |
| 10 | Method of approach | How were participants approached? e*.g. face-to-face, telephone, mail, email* |  |  |
| 9 | Sample size | How many participants were in the study? |  |  |
| 9 | Non-participation | How many people refused to participate or dropped out? Reasons? |  |  |
| Setting |  |  |  |  |
| 10-11 | Setting of data collection | Where was the data collected? e*.g. home, clinic, workplace* |  |  |
| 10-11 | Presence of non-participants | Was anyone else present besides the participants and researchers? |  |  |
| 9-10 | Description of sample | What are the important characteristics of the sample? *e.g. demographic data, date* |  |  |
| Data collection |  |  |  |  |
| 12 | Interview guide | Were questions, prompts, guides provided by the authors? Was it pilot tested? |  |  |
| no | Repeat interviews | Were repeat interviews carried out? If yes, how many? |  |  |
| 11 | Audio/visual recording | Did the research use audio or visual recording to collect the data? |  |  |
| 11 | Field notes | Were field notes made during and/or after the interview or focus group? |  |  |
| 11 | Duration | What was the duration of the interviews or focus group? |  |  |
| 7 | Data saturation | Was data saturation discussed? |  |  |
| 11-14 | Transcripts returned | Were transcripts returned to participants for comment and/or correction? |  |  |
| **Domain 3: analysis and findings**z |  |  |  |  |
| Data analysis |  |  |  |  |
| 12-13 | Number of data coders | How many data coders coded the data? |  |  |
| 16 | Description of the coding tree | Did authors provide a description of the coding tree? |  |  |
| 16-23 | Derivation of themes | Were themes identified in advance or derived from the data? |  |  |
| 13 | Software | What software, if applicable, was used to manage the data? |  |  |
| 14 | Participant checking | Did participants provide feedback on the findings? |  |  |
| Reporting |  |  |  |  |
| 16-23 | Quotations presented | Were participant quotations presented to illustrate the themes / findings? Was each quotation identified? e*.g. participant number* |  |  |
